# Supplementary material for: Clonal cell states link gastroesophageal junction tissues with metaplasia and cancer
Source: Nat Commun. 2025 Dec 8;16:10952. doi: 10.1038/s41467-025-66302-w (PMC12686426; doi:10.1038/s41467-025-66302-w)
Supplement: Supplementary file 1 — Supplementary Information [file 41467_2025_66302_MOESM1_ESM.pdf]

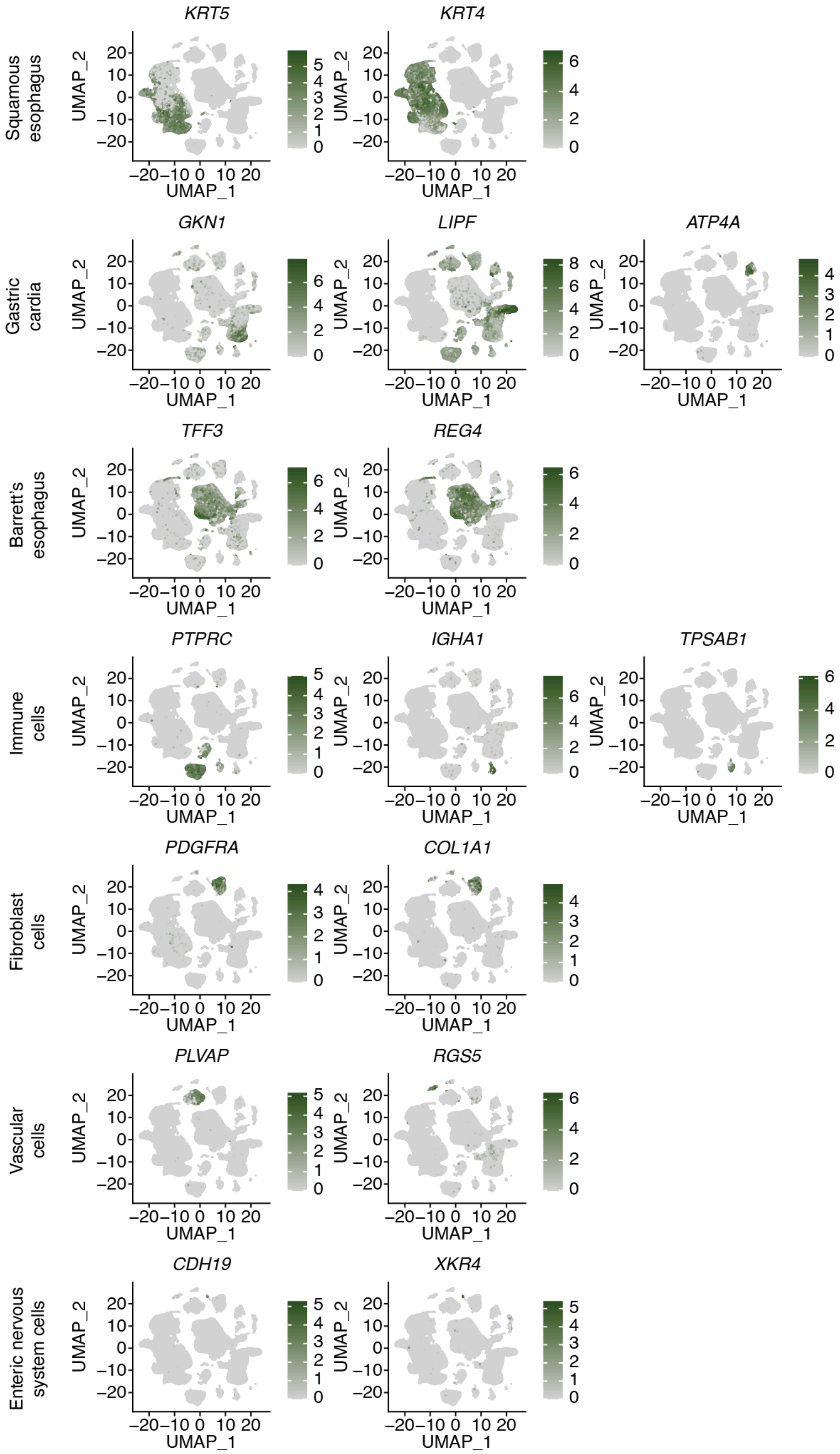

Fig. S1. Annotation of GEJ tissues and supporting cell populations. UMAPs of scRNA-seq of all the samples collected in this study featuring expression of high-level tissue markers of the normal esophagus (*KRT5*, *KRT4*), the gastric cardia (*GKN1*, *LIPF*, *ATP4A*), Barrett's esophagus (*TFF3, REG4*), immune cells (*PTPRC*, *IGHA1*, *TPSAB1*), fibroblasts (*PDGFRA*, *COL1A1*), vascular cells (*PLVAP*, *RGS5*), and enteric nervous system cells (*CHD19*, *XKR4*).

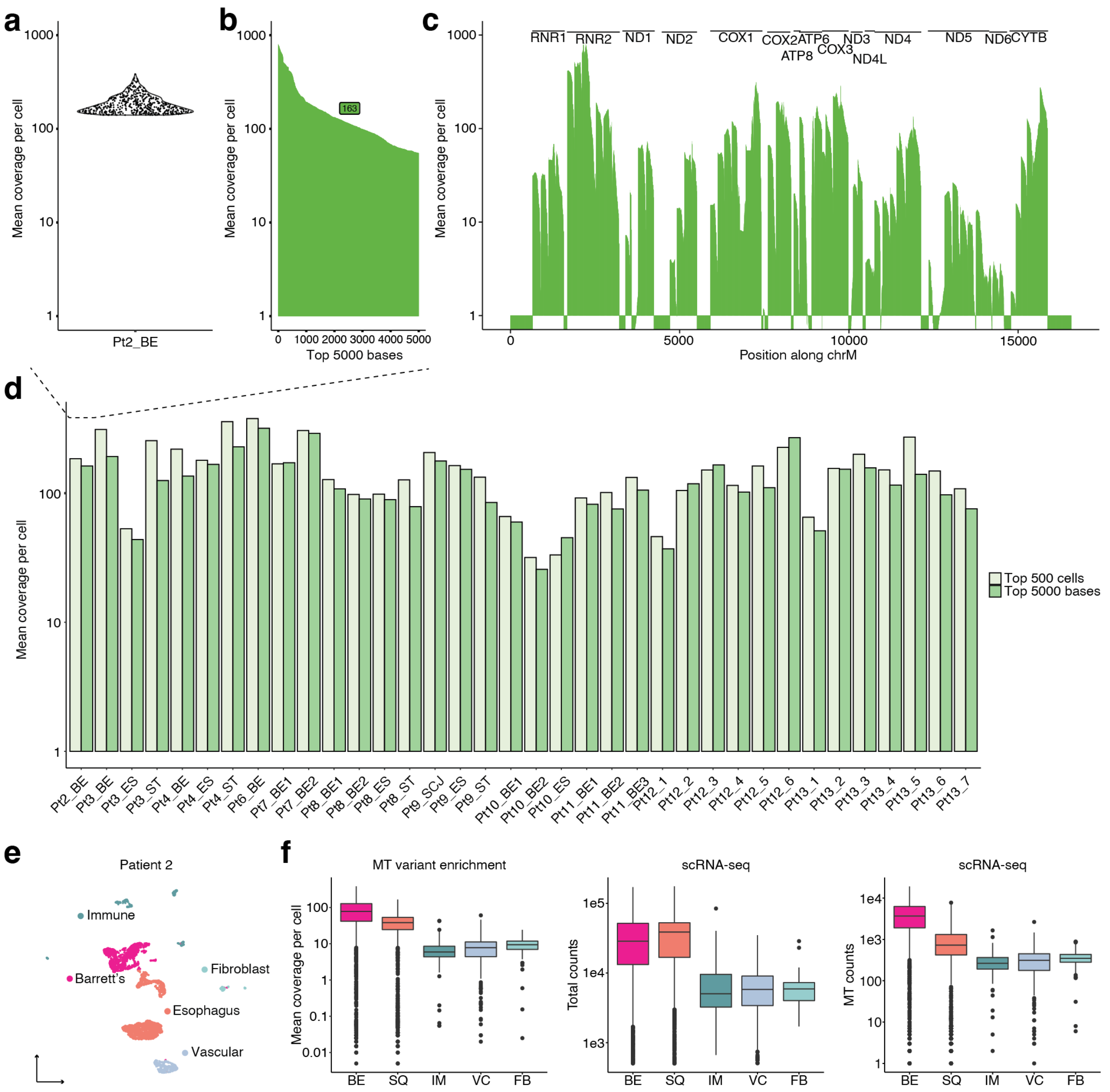

Fig. S2. Mitochondrial variant enrichment sequencing coverage. (**a**) Sina plot of the mean coverage across the mitochondrial genome for the top 500 cells in the Barrett's esophagus sample from Patient 2. (**b**) Barplot of the mean coverage per cell at the top 5000 bases in the Barrett's esophagus sample from Patient 2. (**c**) Barplot of mean coverage across the mitochondrial genome for all cells in the Barrett's esophagus sample from Patient 2. (**d**) Grouped barplot of mean coverage for the top 500 cells and top 5000 bases in each sample analyzed by mitochondrial variant enrichment in this study. (**e**) UMAP of scRNA-seq of a single biopsy from Patient 2 that contained Barrett's esophagus and squamous esophagus cells; panels (a)-(c) and (f) describe this sample. (**f**) Left: Boxplot of the mean mitochondrial variant enrichment coverage for all cells in the sample from Patient 2 split by cell type. Middle: Boxplot of the total RNA counts from scRNA-seq of the same cells split by cell type. Right: Boxplot of the mitochondrial subset of RNA counts from scRNA-seq of the same cells split by cell type. In all boxplots, the center line represents the median, box bounds represent the first and third quartiles (25th and 75th percentiles), whiskers extend to the minima and maxima within 1.5 times the interquartile range, and points beyond whiskers represent outliers.

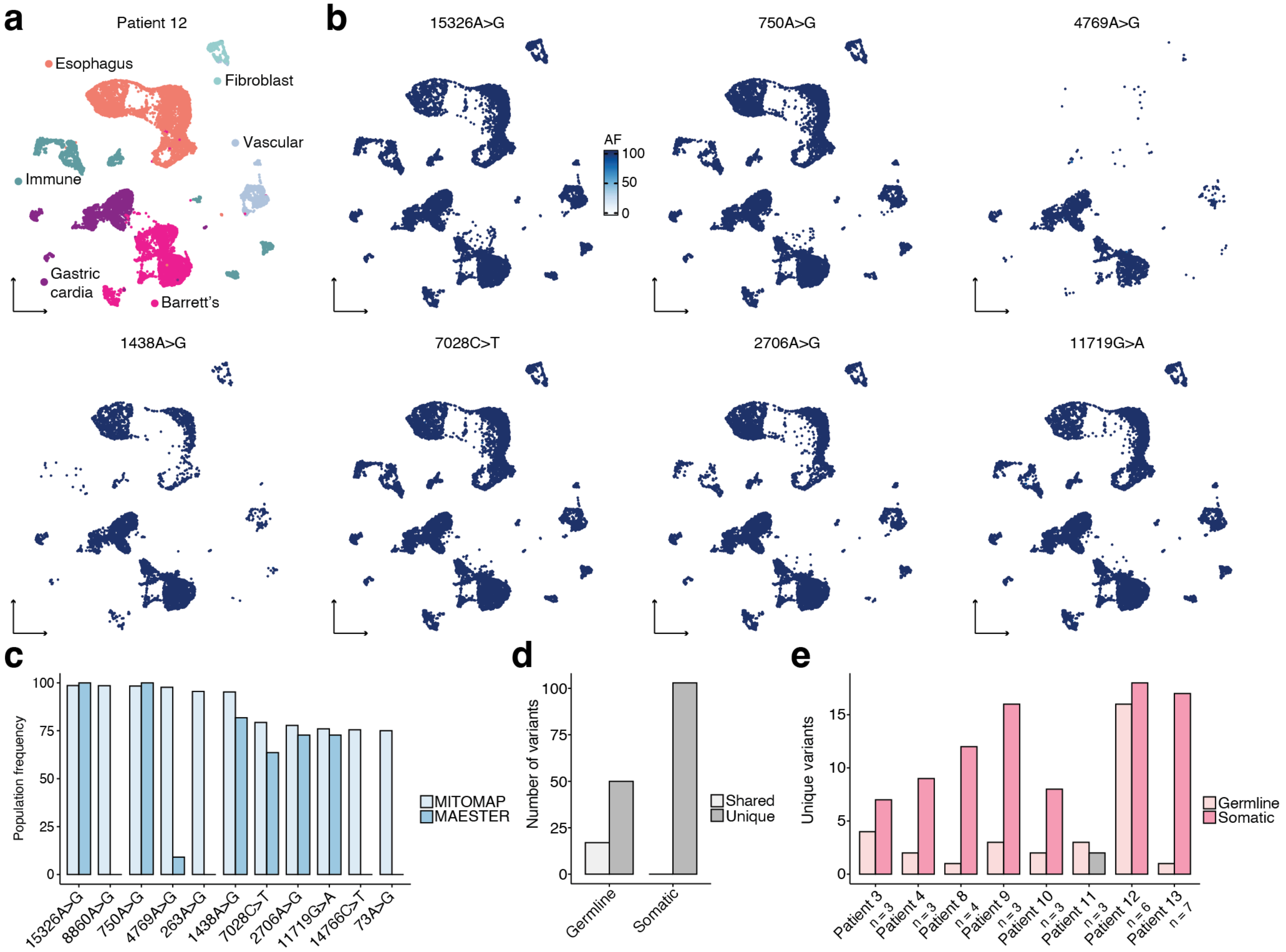

Fig. S3. Detection of germline mtDNA variants and comparison with somatic mutations. (**a**) UMAP of scRNA-seq of all cells from Patient 12. (**b**) UMAPs of (a) colored with the allele frequencies of the seven mitochondrial germline mutations detected in this patient that belong to the set of the 11 most common ancestral mutations. (**c**) Grouped barplot of the frequencies at which the 11 most common ancestral mitochondrial mutations occur in the MITOMAP database and our mitochondrial variant enrichment data (MAESTER). (**d**) Grouped barplot of the number of shared and unique germline and somatic variants in our dataset. (**e**) Grouped barplot of the number of unique germline and somatic variants detected across patients with at least three biopsies that together contained multiple tissues. The number of biopsies for each patient is given by the n.

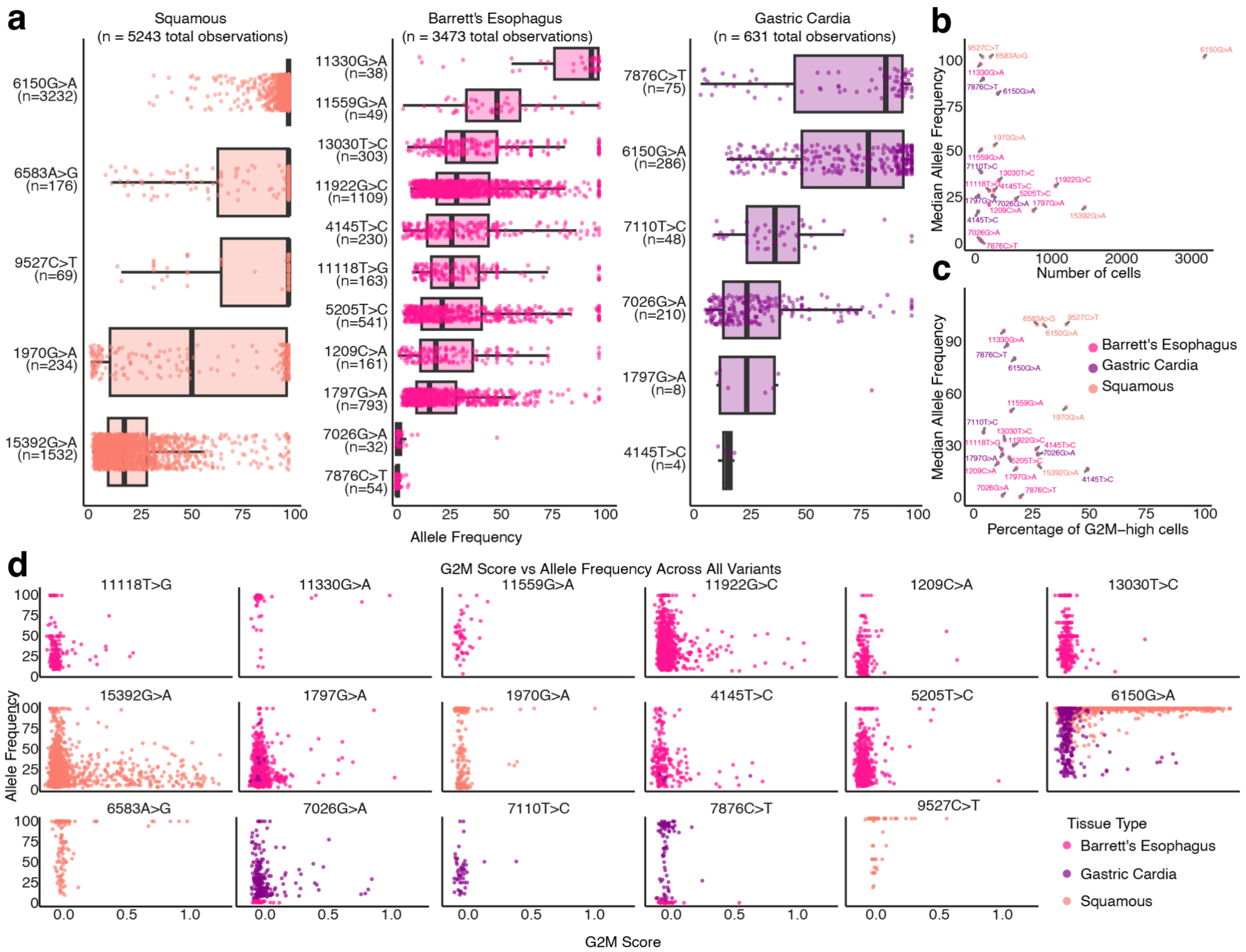

Fig. S4. Analysis of mitochondrial allele frequencies across different tissue types and cell proliferation states. (**a**) Boxplots showing the distribution of allele frequencies for significant mtDNA variants detected in each tissue. (**b**) Scatterplot displaying the relationship between median allele frequency and number of cells for each mtDNA variant. (**c**) Scatterplot showing the relationship between median allele frequency and percentage of G2M-high cells (proliferative cells) within each variant lineage. No clear correlation is observed between allele frequency and proliferation status. (**d**) Single-cell analysis of G2M score versus allele frequency for individual mtDNA variants across all tissues. Each panel shows a different mutation, with points colored by tissue type as indicated in the legend. In all boxplots, the center line represents the median, box bounds represent the first and third quartiles (25th and 75th percentiles), whiskers extend to the minima and maxima within 1.5 times the interquartile range. All points are plotted on top of boxplots.

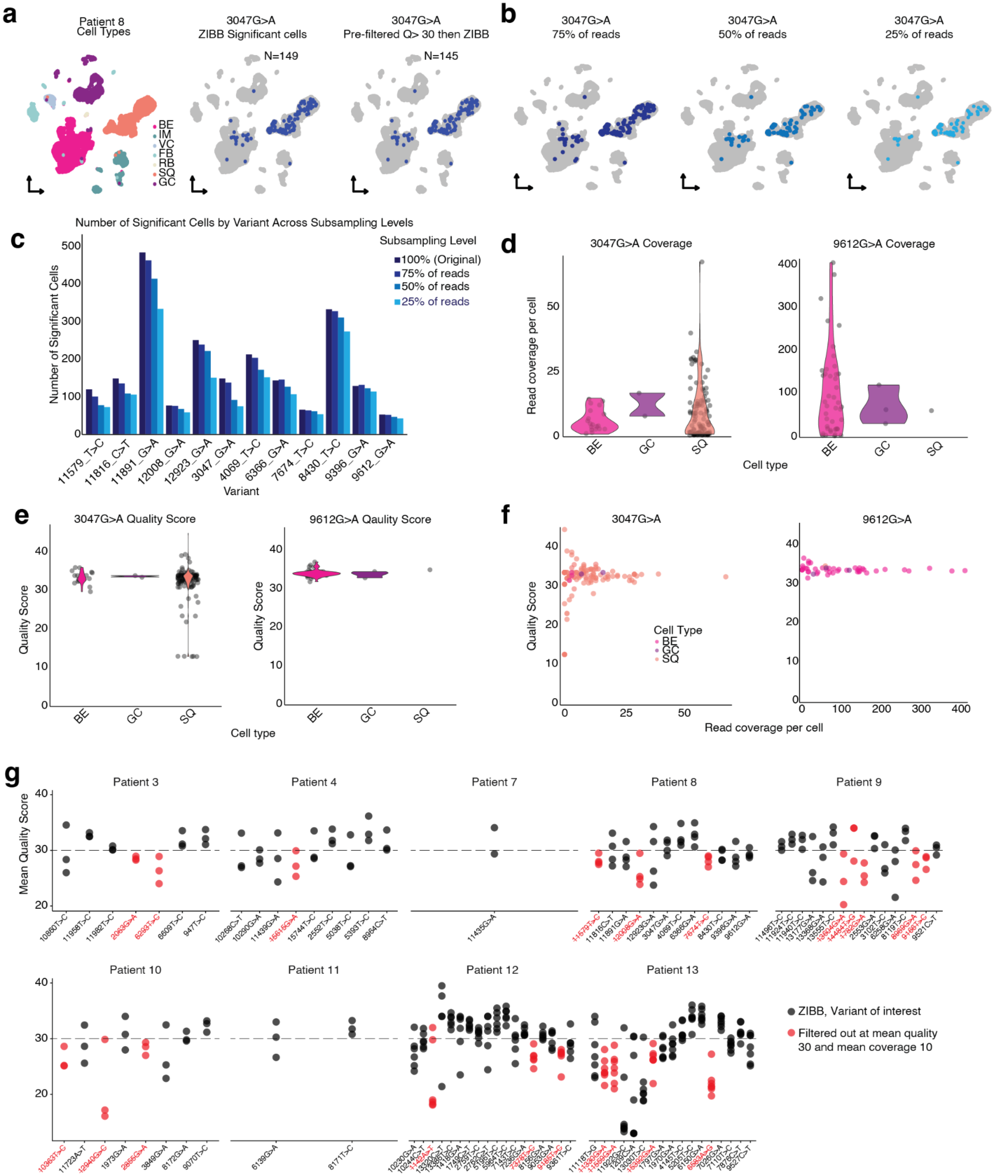

Fig. S5. Quality control and filtering of mitochondrial variants used for lineage tracing. (**a**) We tested the effects of pre-filtering quality > 30 for variants prior to ZIBB analysis for Patient 8. First plot is UMAP visualization of cell types from Patient 8 (left) colored by tissue identity. Next UMAP plot shows the significant cells for 3047G>A using ZIBB without pre-filtering. Final UMAP plot shows significant cells when pre-filtering for reads with quality scores > 30 for the variant. (**b**) Summary plots from sensitivity analysis in which we

subsampled the sequencing data down to 75%, 50%, and 25% of the initial coverage and then ran the ZIBB model to identify variant positive cells for 3047G>A. UMAP plots show significant 3047G>A cells at the different subsampled levels. (**c**) Barplot summarizing subsampling results with the total number of significant cells for each variant in Patient 8. The different colored bars represent the different subsampling levels. (**d**) Coverage distribution of variants 3047G>A (top) and 9612G>A (bottom) across cell types. (**e**) Quality score distributions for variants 3047G>A and 9612G>A by cell type. (**f**) Relationship between quality scores and coverage for variants 3047G>A and 9612G>A across different cell types. (**g**) Mean quality scores for mitochondrial variants across patients. Our filtering workflow identifies variants of interest based on mean quality > 27, mean coverage > 5, and allele frequency of > 25% in at least 1% of epithelial cells. These variants are plotted. Red points are variants that would be removed if the threshold was set at mean quality > 30 and mean coverage > 10.

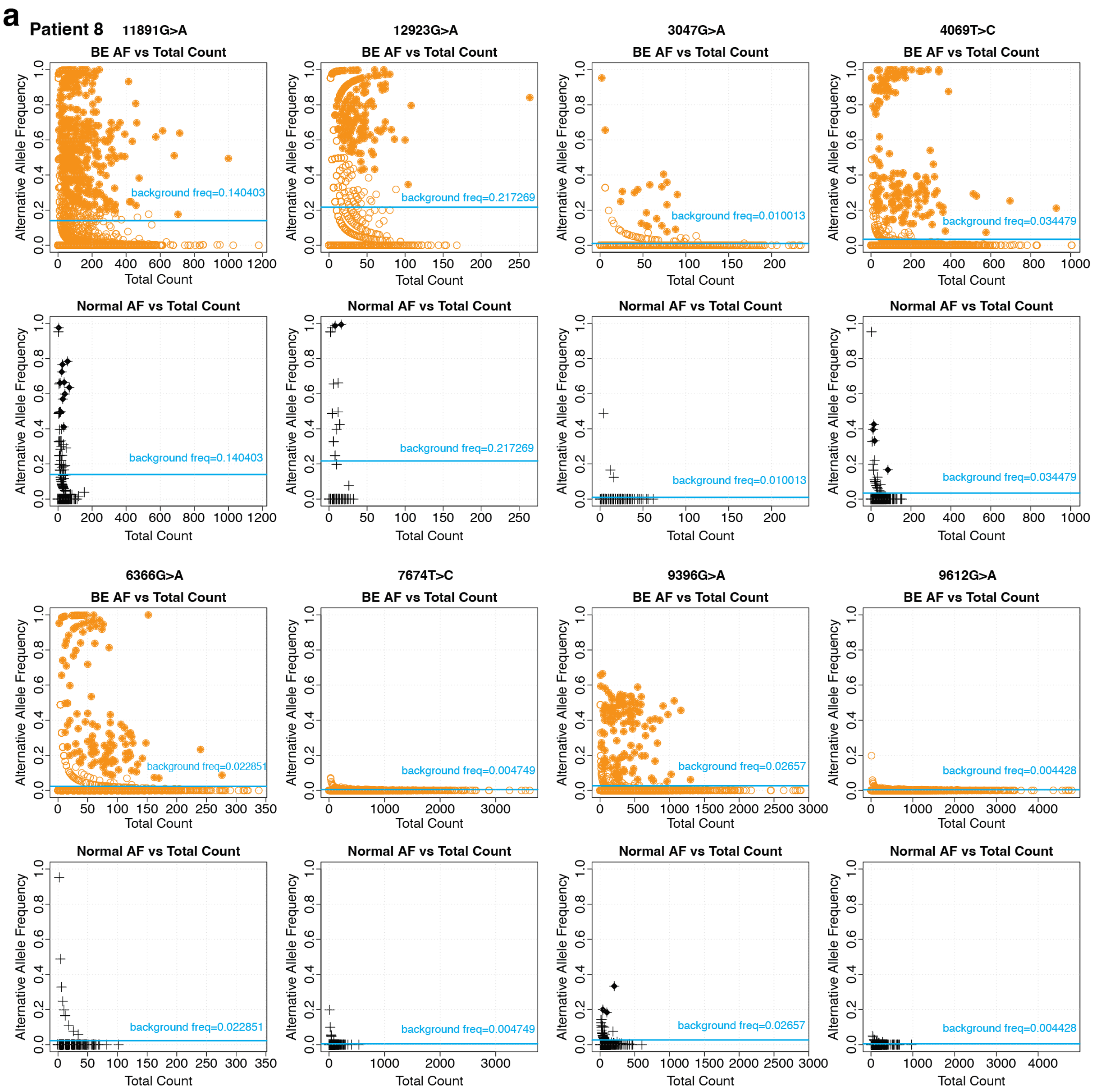

Fig. S6. Assessment of mitochondrial variant detection using the ZIBB model in Patient 8. (a) Scatter plots comparing alternative allele frequency versus total read count for eight different mtDNA variants. Top panels display Barrett's esophagus cells (orange dots), with filled circles indicating cells with statistically significant variant detection and open circles showing cells below significance threshold. Bottom panels show corresponding plots for normal control cells (black crosses). Green horizontal lines indicate the estimated background frequency for each variant as determined by the ZIBB statistical model.

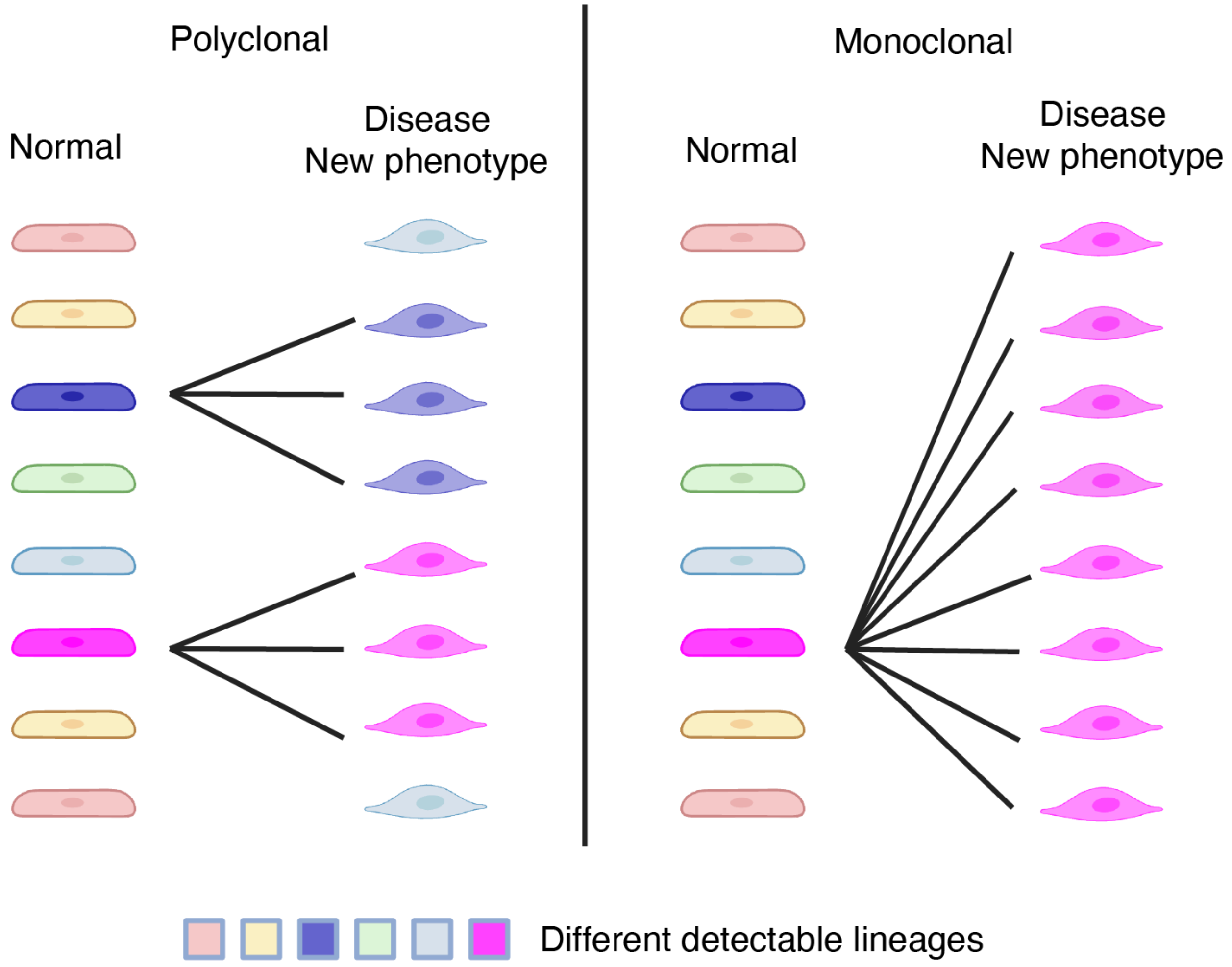

Fig. S7 Polyclonal versus monoclonal models of disease development tracked by lineage markers. Schematic illustrating how lineage markers (represented by different colors) reveal distinct modes of tissue transformation. Polyclonal model where multiple cells independently acquire a disease phenotype (e.g., Barrett's esophagus). Different lineages each contain cells that have independently transformed to the disease state, resulting in phenotypic uniformity despite diverse origins. Monoclonal model where a single cell acquires the disease phenotype and transmits it to all descendants (e.g., dysplasia). All disease cells share both the lineage marker and the inherited pathological phenotype. Normal cells that did not transform retain their original phenotypes.

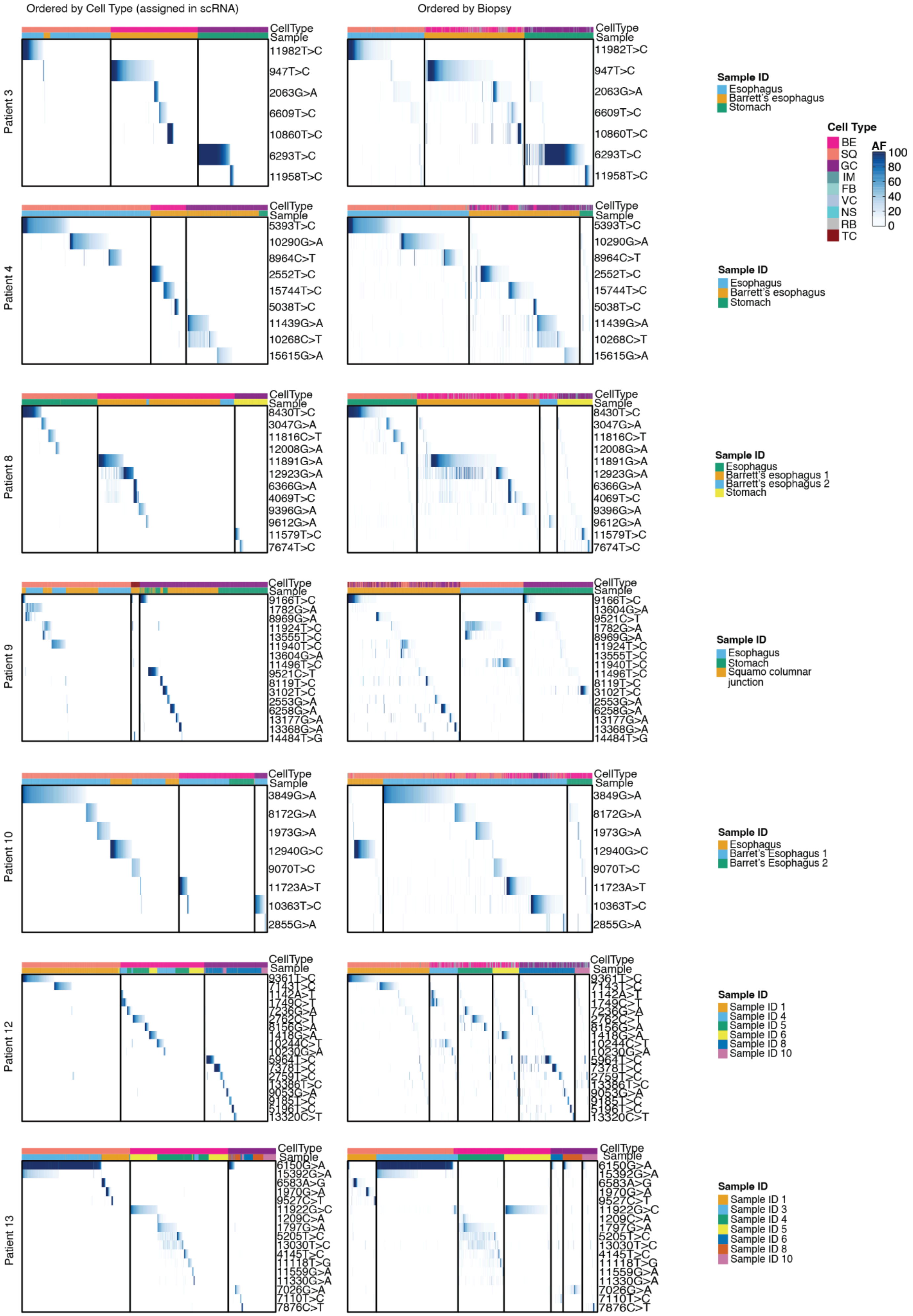

Ordered by Cell Type (assigned in scRNA)
Ordered by Biopsy
CellType
Sample
Patient 3
11982T>C
947T>C
2063G>A
6609T>C
10860T>C
6293T>C
11958T>C
Sample ID
Esophagus
Barrett's esophagus
Stomach
Cell Type
BE
SQ
GC
IM
FB
VC
NS
RB
TC
AF
100
80
60
40
20
0
Patient 4
5393T>C
10290G>A
8964C>T
2552T>C
15744T>C
5038T>C
11439G>A
10268C>T
15615G>A
Sample ID
Esophagus
Barrett's esophagus
Stomach
Patient 8
8430T>C
3047G>A
11816C>T
12008G>A
11891G>A
12923G>A
6366G>A
4069T>C
9396G>A
9612G>A
11579T>C
7674T>C
Sample ID
Esophagus
Barrett's esophagus 1
Barrett's esophagus 2
Stomach
Patient 9
9166T>C
1782G>A
8969G>A
11924T>C
13555T>C
11940T>C
13604G>A
11496T>C
9521C>T
8119T>C
3102T>C
2553G>A
6258G>A
13177G>A
13368G>A
14484T>G
Sample ID
Esophagus
Stomach
Squamo columnar junction
Patient 10
3849G>A
8172G>A
1973G>A
12940G>C
9070T>C
11723A>T
10363T>C
2855G>A
Sample ID
Esophagus
Barret's Esophagus 1
Barret's Esophagus 2
Patient 12
9361T>C
7143T>C
1142A>T
1749C>T
7236G>A
2762C>T
8156G>A
14183G>A
10244C>T
10230G>A
5964T>C
7378T>C
2759T>C
13386T>C
9053G>A
9185T>C
5196T>C
13320C>T
Sample ID
Sample ID 1
Sample ID 4
Sample ID 5
Sample ID 6
Sample ID 8
Sample ID 10
Patient 13
6150G>A
15392G>A
6583A>G
1970G>A
9527C>T
11922G>C
1209C>A
1797G>A
5205T>C
13030T>C
4145T>C
11118T>G
11559G>A
11330G>A
7026G>A
7110T>C
7876C>T
Sample ID
Sample ID 1
Sample ID 3
Sample ID 4
Sample ID 5
Sample ID 6
Sample ID 8
Sample ID 10

Fig. S8. Somatic mtDNA mutations in patients with cells from multiple epithelial tissues. Heatmaps show the allele frequencies of mtDNA mutations within the cells of patients with biopsies from multiple gastroesophageal junction tissues. Cells are grouped by tissue type in the left column and by biopsy in the right column. In the left column, cells without variants are included. In the right column, cells without variants are not included to allow better visualization. Cell types are indicated by colors at the top of each heatmap (BE = Barrett's esophagus, SQ = squamous esophagus, GC = gastric cardia, IM = immune, FB = fibroblast, VS = vascular, NS = not specified). SampleIDs are color-coded.

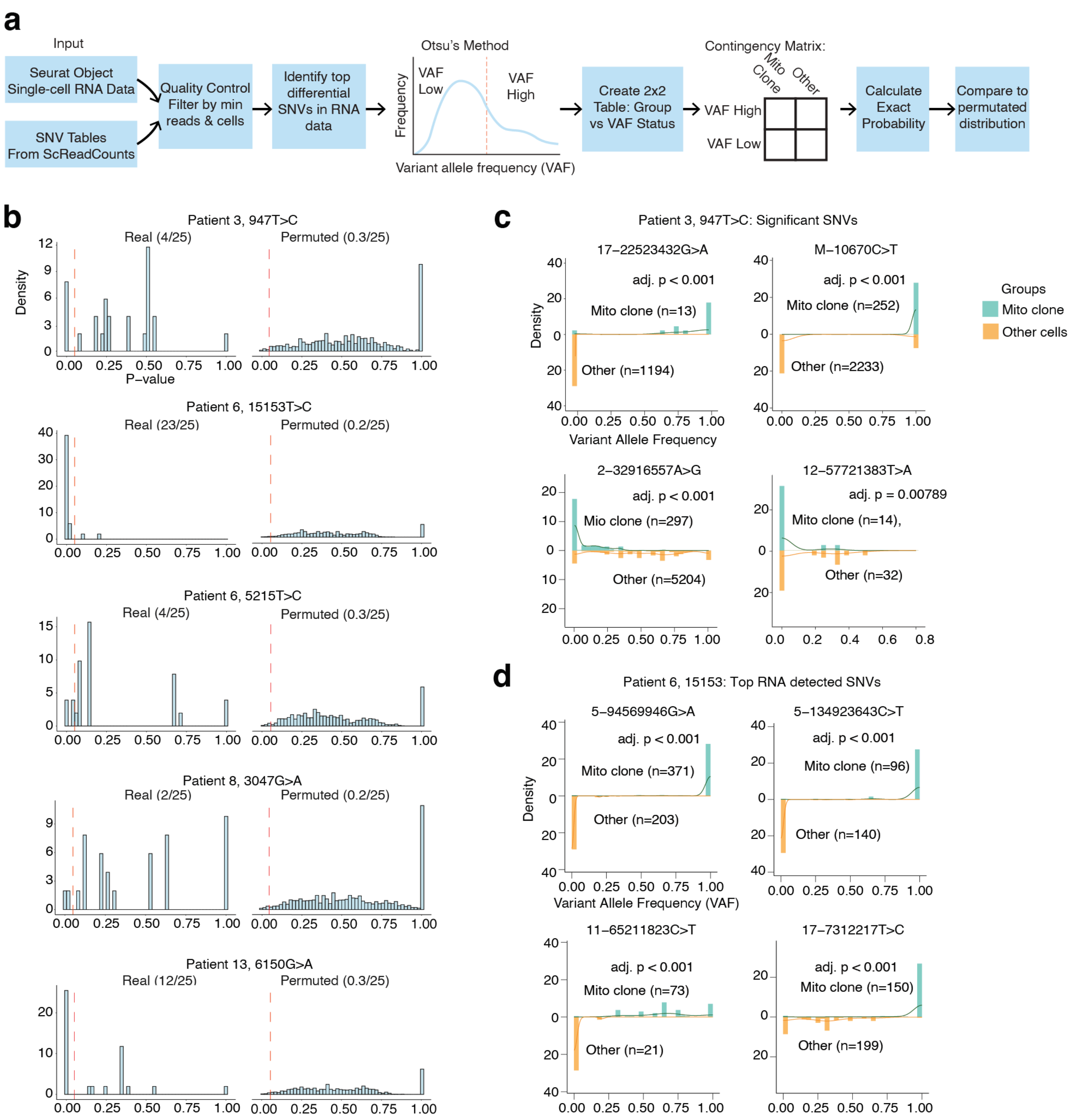

Fig. S9. Validation of mitochondrial variant-based lineage assignments using RNA detected single nucleotide variants (SNVs). (**a**) Schematic workflow of the SNV analysis pipeline. (**b**) Density histograms showing distributions of p-values from Fisher's exact tests for real data versus permuted data across multiple patients and mitochondrial variants. Enrichment of low p-values in real data indicates significant associations between mitochondrial variants and RNA detected SNV patterns. (**c**) Variant allele frequency distributions for representative SNVs in Patient 3, showing significant differences between cells within mitochondrial lineages (teal) and other cells (orange). (**d**) VAF distributions for top RNA-detected SNVs in Patient 6 (15153 lineage), demonstrating concordant SNV patterns that support mitochondrial variant-based lineage assignments.

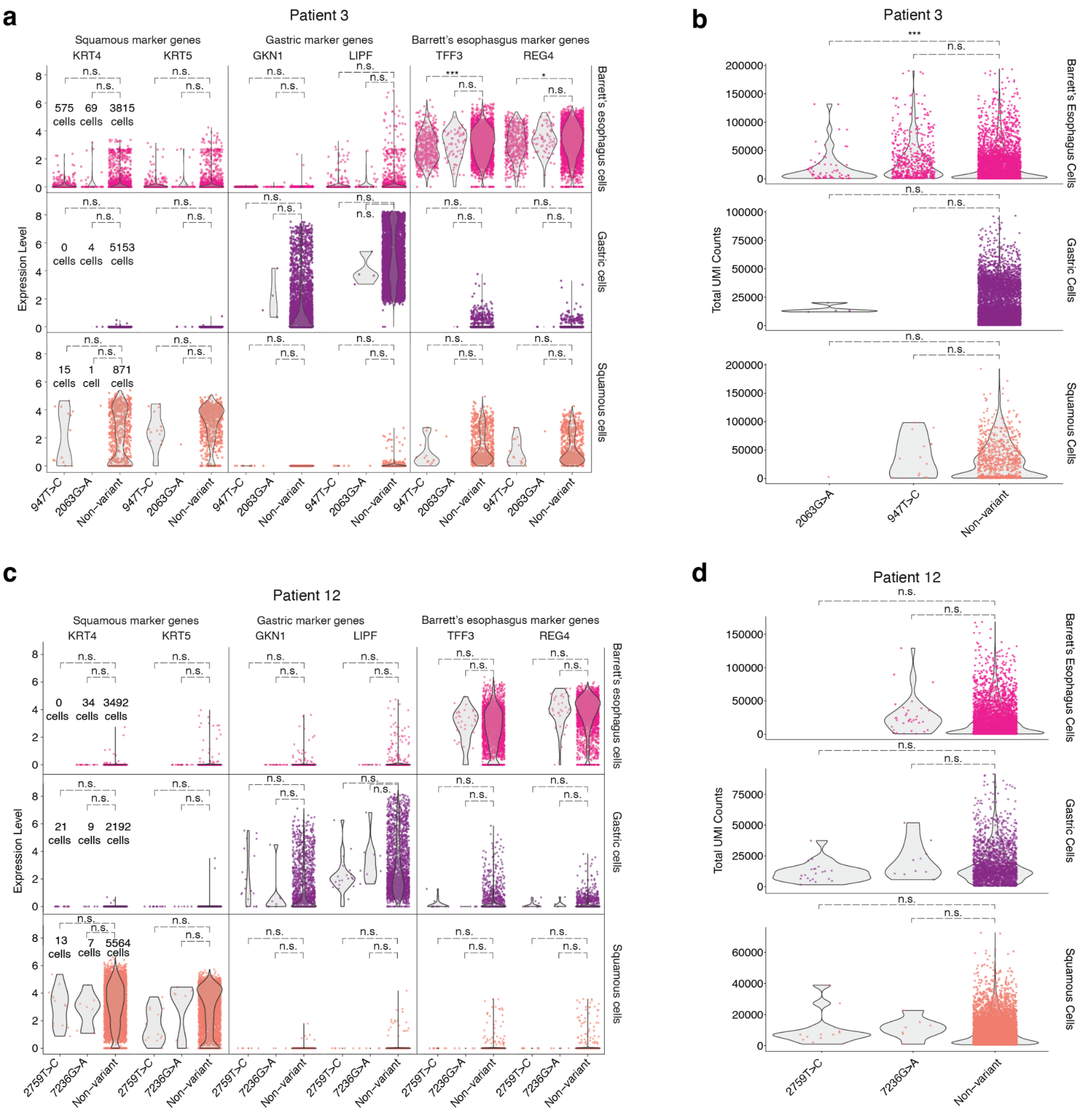

Fig. S10. Validation that cells containing cross-tissue mitochondrial variants are not doublet artifacts. (**a**) Violin plots of data from Patient 3 showing expression levels of canonical marker genes for squamous epithelium (*KRT4*, *KRT5*), gastric cardia (*GKN1*, *LIPF*), and Barrett's esophagus (*TFF3*, *REG4*) in cells with specific mitochondrial variants (947T>C and 2605G>A) compared to non-variant cells of the same tissue type. Top row shows Barrett's esophagus cells, middle row shows gastric cells, and bottom row shows squamous cells. Cell numbers for each group are indicated below each plot. (**b**) Distribution of total UMI counts in cells containing the same mitochondrial variants as in (a), demonstrating that variant-containing cells have UMI distributions comparable to non-variant cells of the same tissue type, rather than the abnormally high UMI counts typically observed in cell doublets. (**c**) Violin plots of data from Patient 12 showing expression levels of

the same canonical marker genes as in (a) in cells with specific mitochondrial variants (2759T>C and 7236G>A) compared to non-variant cells. (**d**) Distribution of total UMI counts in cells containing the same mitochondrial variants as in (c) for Patient 12, demonstrating comparable UMI distributions between variant-containing and non-variant cells. Statistical significance was assessed using two-sided Wilcoxon rank-sum tests with Benjamini-Hochberg correction for multiple comparisons (n.s. = not significant, $p > 0.05$; * $p < 0.05$; ** $p < 0.01$; *** $p < 0.001$).

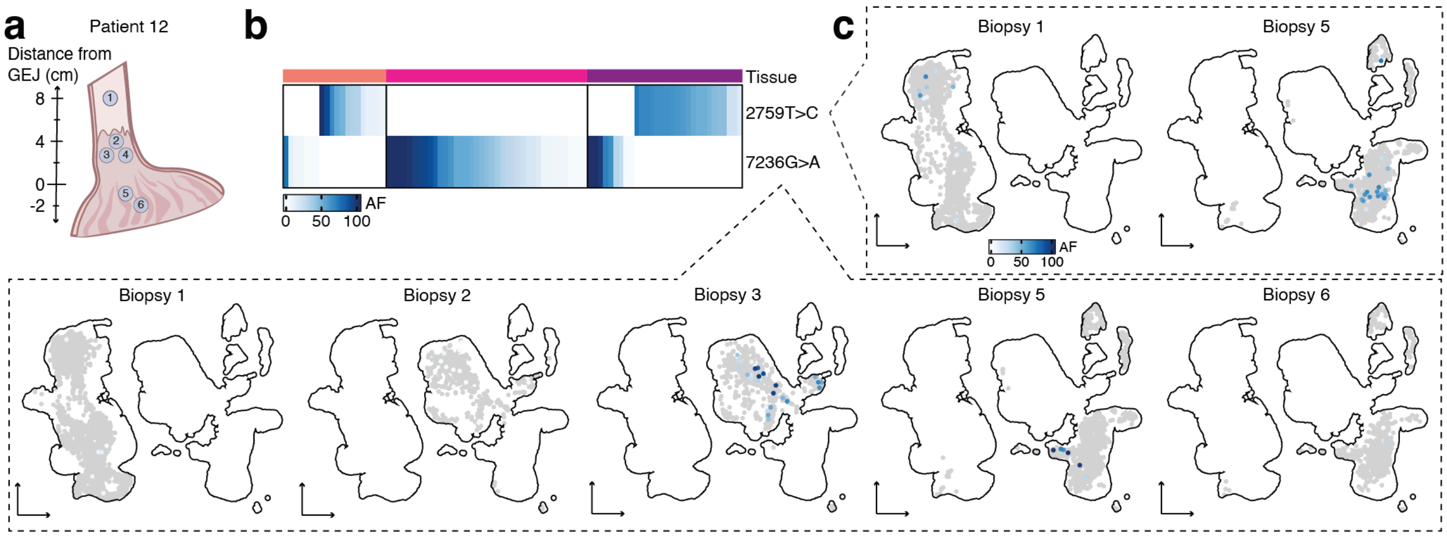

Fig. S11. mtDNA mutations detected in multiple epithelial tissues from Patient 12. (**a**) Diagram of the gastroesophageal junction region labeled with the locations of the biopsies from Patient 12. Cells are grouped by tissue type, which is labeled according to the colors used in the UMAP in Fig. 1C. (**b**) Heatmap showing the allele frequency of mtDNA mutations 2759T>C and 7236G>A in squamous esophagus, Barrett's esophagus, and gastric cardia cells from patient 12. (**c**) UMAPs of scRNA-seq of biopsies from Patient 12 containing cells that acquired the mutations 2759T>C and 7236G>A, plotted within the UMAP of all epithelial cells and colored with the allele frequency of the given mutation.

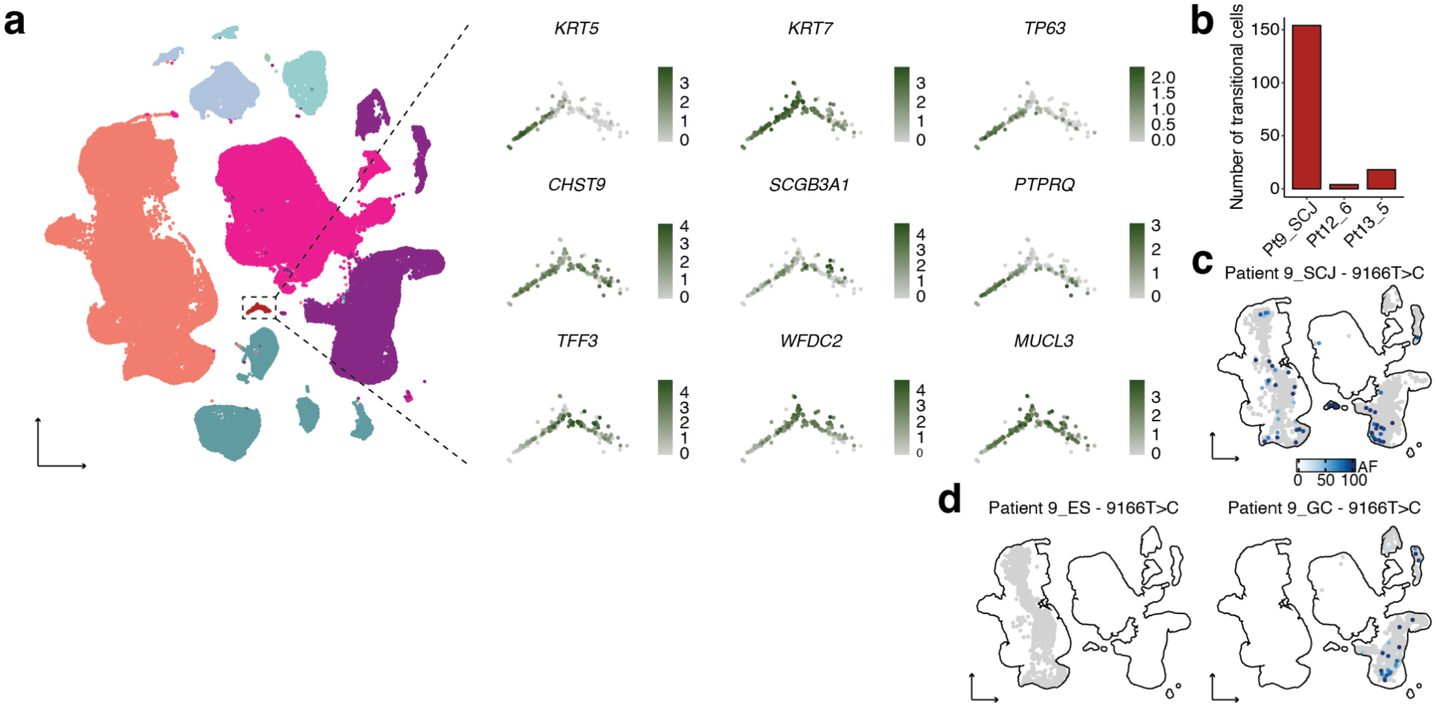

Fig. S12. Transitional basal progenitor cells at the gastroesophageal junction. (**a**) Callout of the transitional basal progenitor cells in the UMAP of all samples showing known (KRT5, KRT7, TP63) and new marker genes. (**b**) Barplot of the breakdown of transitional basal progenitor cells across three samples. (**c**) UMAP of scRNA-seq of the squamocolumnar junction biopsy from Patient 9 containing cells that acquired the mutation 9166T>C, plotted within the UMAP of all epithelial cells and colored with the allele frequency of 9166T>C. (**d**) UMAP of scRNA-seq of two additional samples from Patient 9, collected from the esophagus and gastric cardia. The cells of each biopsy are visualized on the UMAP and colored with the allele frequency of 9166T>C.

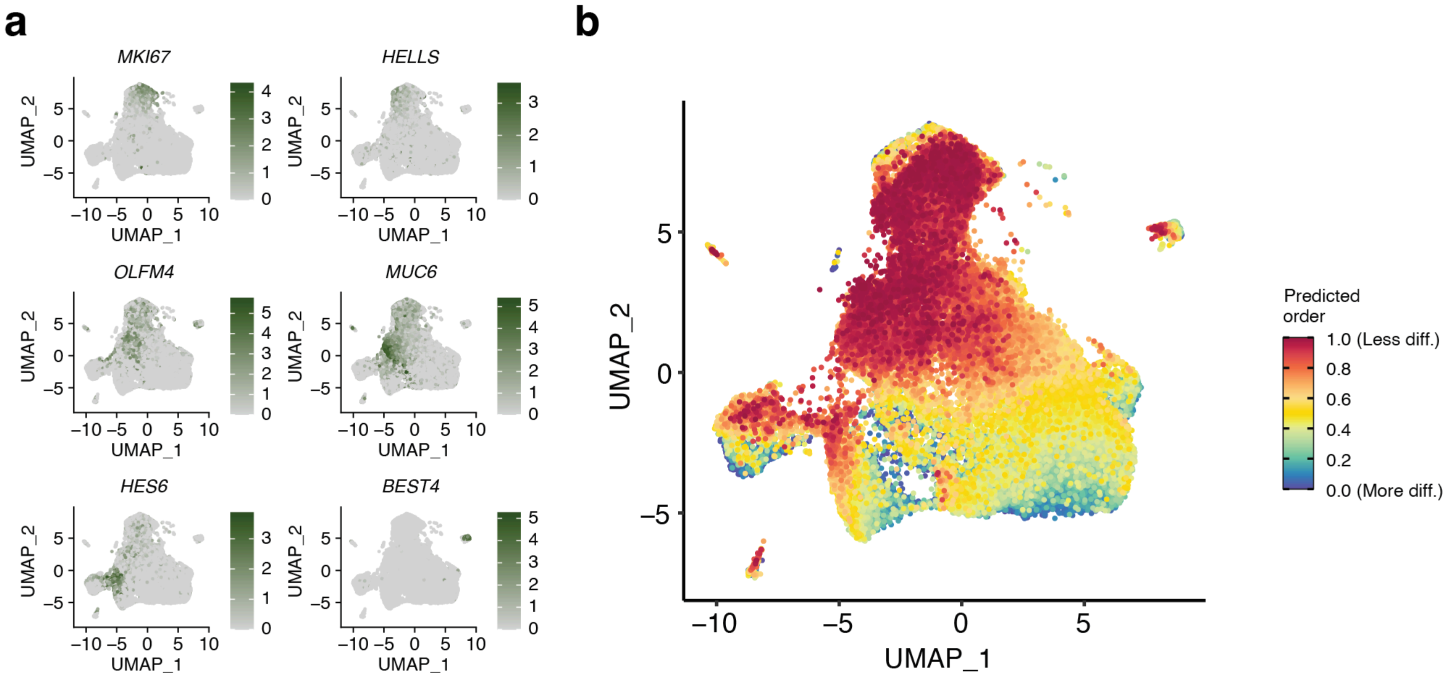

Fig. S13. Markers of proliferative and immature Barrett's esophagus cell types and their differentiation state. (**a**) UMAPs of scRNA-seq of consensus Barrett's esophagus cells featuring the expression of key proliferative (*MKI67*, *HELLS*), progenitor (*OLFM4, MUC6, HES6*), and rare cell (*BEST4*) markers. (**b**) UMAP of scRNA-seq of consensus Barrett's esophagus cells colored by CytoTRACE analysis results, where cells are ordered along a differentiation continuum.

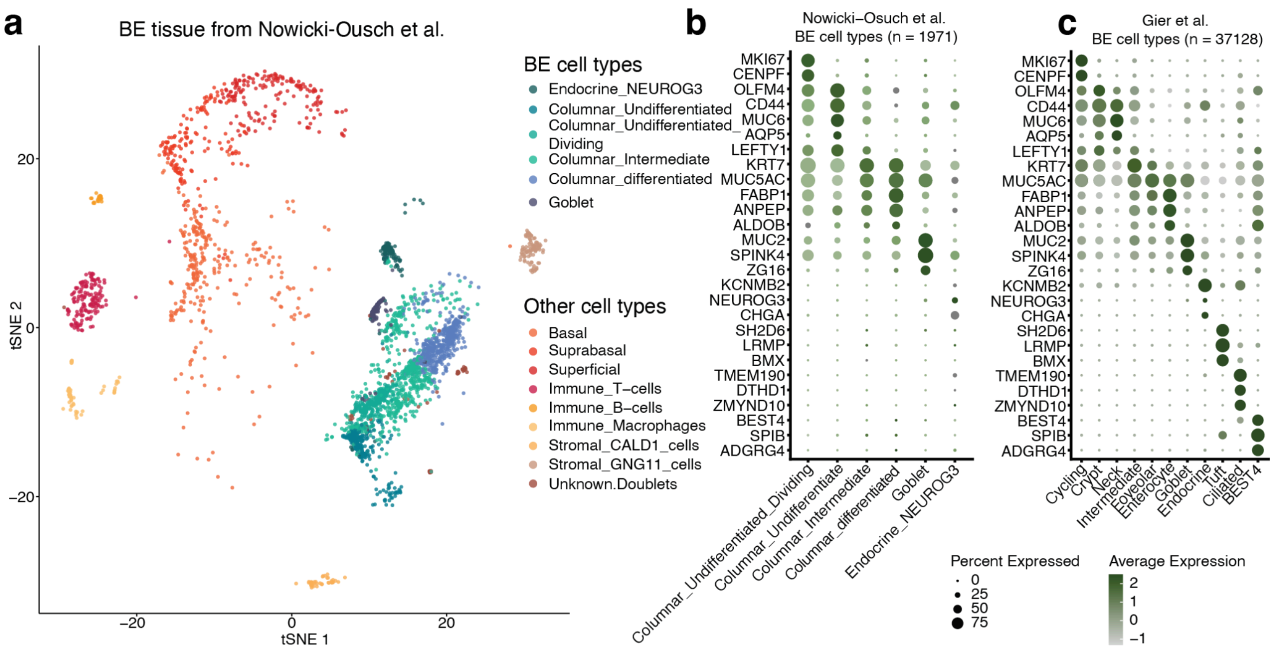

d

Cell types in each study

Nowicki-Osuch et al.	Gier et al.
Columnar_differentiated	Foveolar, Enterocyte
Columnar_Intermediate	Intermediate
Goblet	Goblet
Columnar_Undifferentiated_Dividing	Cycling
Endocrine_NEUROG3	Endocrine
Columnar_Undifferentiated	Crypt, Neck
	BEST4
	Tuft
	Ciliated

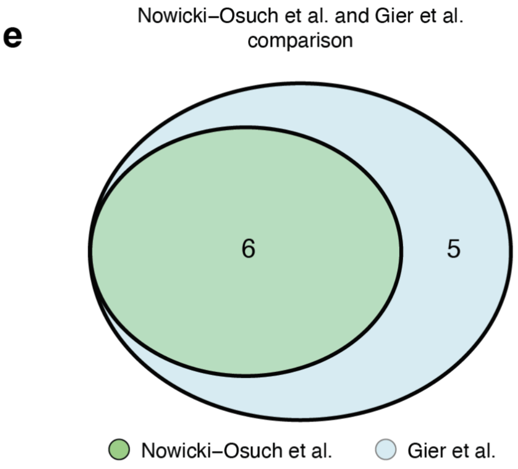

Figure S14. Comparison of Barrett's esophagus cell types between Nowicki-Osuch et al. and our study. (a) tSNE visualization of Barrett's esophagus cell types from Nowicki-Osuch et al., colored by their original cell type annotations. **(b)** Bubble plot showing marker gene expression across cell types identified by Nowicki-Osuch et al. (n = 1,971 cells). **(c)** Bubble plot showing the same marker genes across our BE cell types (n = 37,128 cells), demonstrating both shared and novel populations. **(d)** Table mapping cell type terminology between the two studies, highlighting the six shared cell populations and three novel cell types (BEST4, Tuft, and Ciliated) identified in our study. **(e)** Venn diagram quantifying the overlap in cell types between studies, showing six shared cell types and three additional cell types identified in our larger dataset.

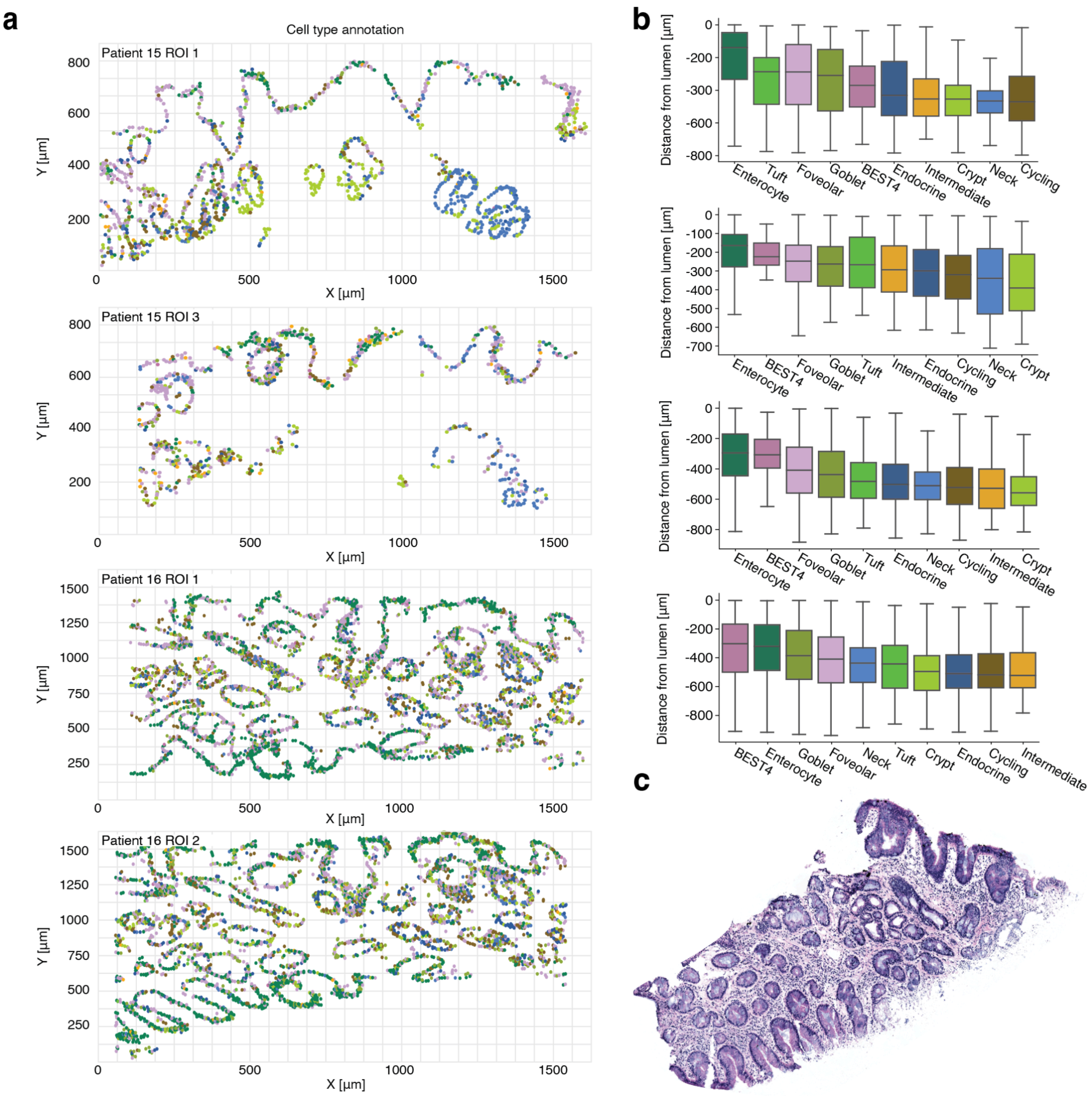

Fig. S15. Spatial location of Barrett's esophagus cell types. (**a**) Sections of fresh frozen Barrett's esophagus tissue from multiple patients with cell type annotations from scRNA-seq integrated with seqFISH data; the colors match the labels in (b). (**b**) Boxplots of distance to the lumen for all epithelial cells in the corresponding section in (a) split by cell type. In all boxplots, the center line represents the median, box bounds represent the first and third quartiles (25th and 75th percentiles), whiskers extend to the minima and maxima within 1.5 times the interquartile range from the quartiles, and outliers were removed. (**c**) Near-adjacent H&E image of the same tissue section for patient 16.

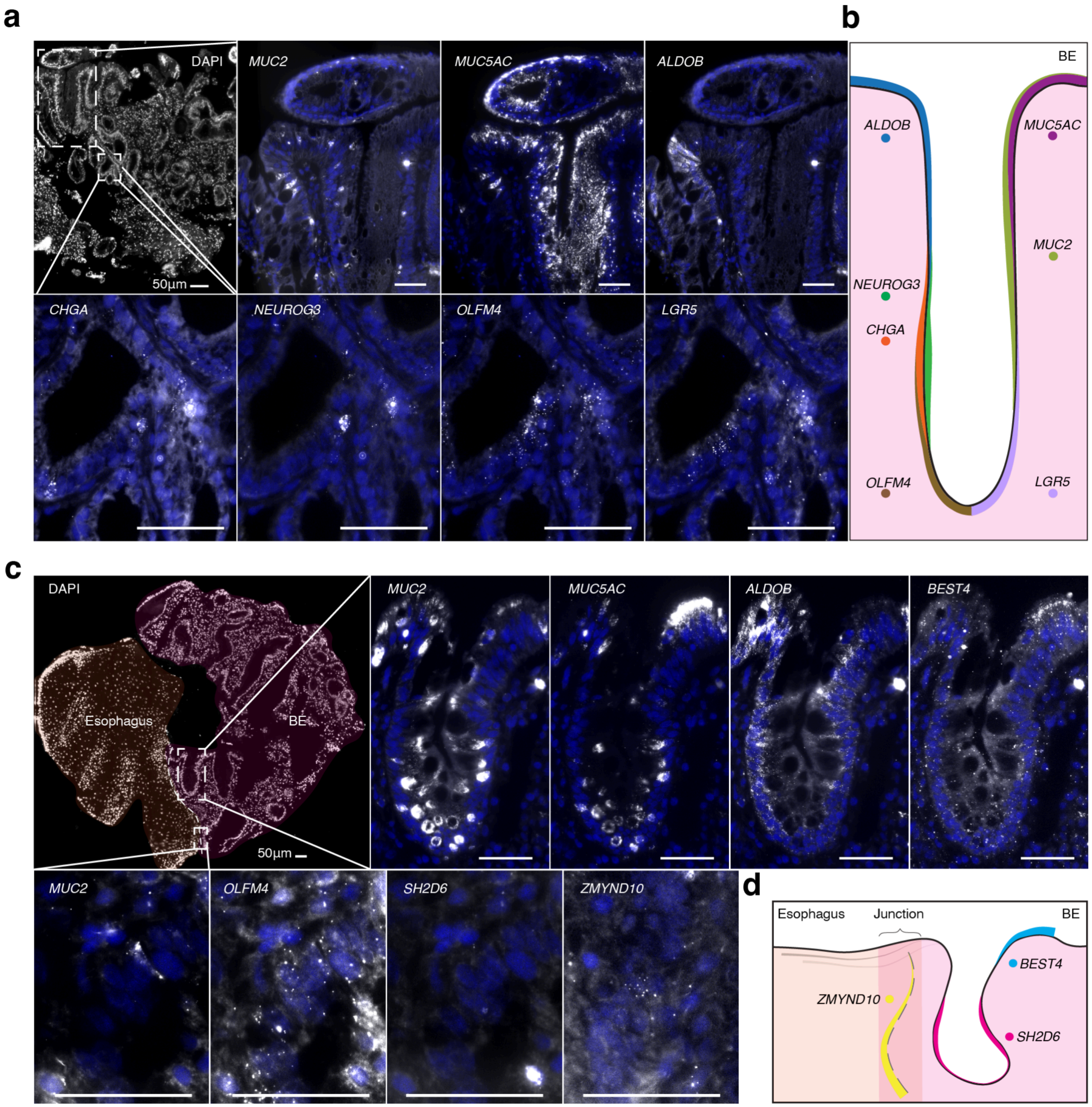

Fig. S16. HCR RNA FISH validation of spatial location of Barrett's esophagus cell types. (**a**) Multiplexed HCR RNA FISH of fresh frozen Barrett's esophagus sections for *MUC2* (goblet), *MUC5AC* (foveolar), *ALDOB* (enterocyte), *CHGA* (enteroendocrine), *NEUROG3* (enteroendocrine progenitor), *OLFM4* (stem), and *LGR5* (stem) with DAPI counterstain. Scale bars, 50 µm. DAPI staining is displayed in blue. All genes were detected in sections from at least two patients. (**b**) Schematic depicting the general location of key marker genes within Barrett's esophagus glands. (**c**) Multiplexed HCR RNA FISH of fresh frozen tissue sections from the junction of Barrett's esophagus and squamous esophagus for markers of newly discovered rare cell types, as well as typical marker genes from (a): *SH2D6* (tuft), *ZMYND10* (ciliated), and *BEST4*. The image of *ZMYND10* was taken from an adjacent section. Scale bars, 50 µm. DAPI staining is displayed in blue. All genes were detected in sections from at least two patients, with the exception of *ZMYND10*. (**d**) Schematic depicting the general location of rare-cell markers within Barrett's esophagus tissue.

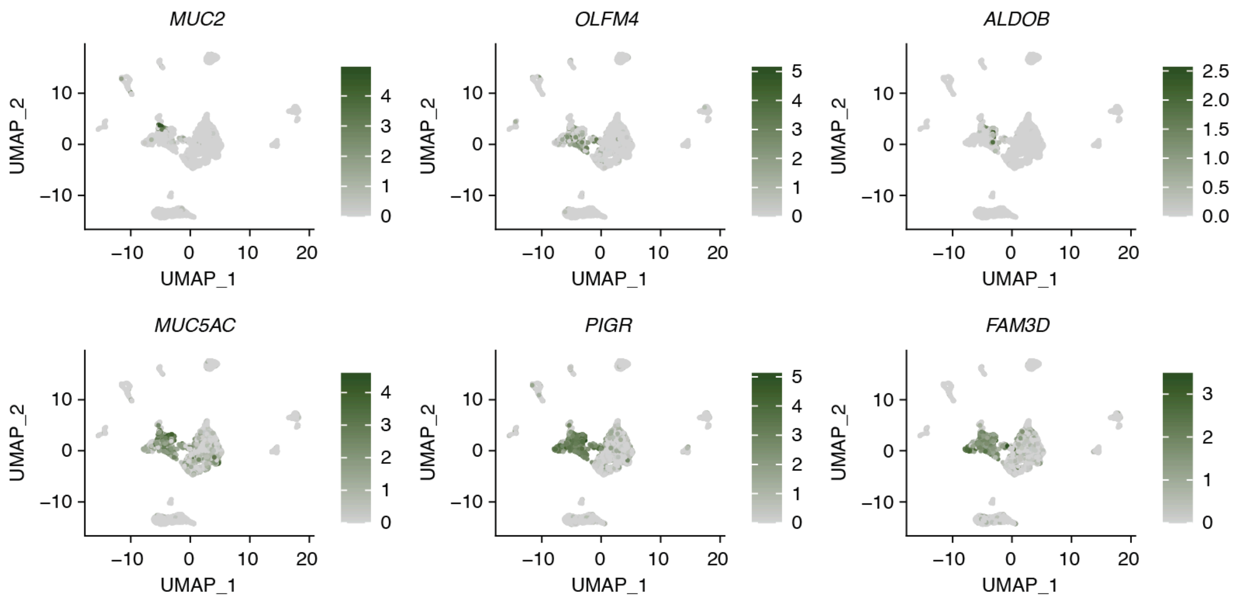

Fig. S17. Markers of typical Barrett's esophagus cell types and barrier integrity genes in dysplastic biopsy. UMAPs of scRNA-seq of cells from a biopsy taken from Patient 6 with high-grade dysplasia examined in depth in Fig. 4 featuring typical Barrett's esophagus cell type markers and barrier integrity genes: *MUC2* (goblet), *OLFM4* (stem), *ALDOB* (enterocyte), *MUC5AC* (foveolar), *PIGR* (barrier), and *FAM3D* (barrier).

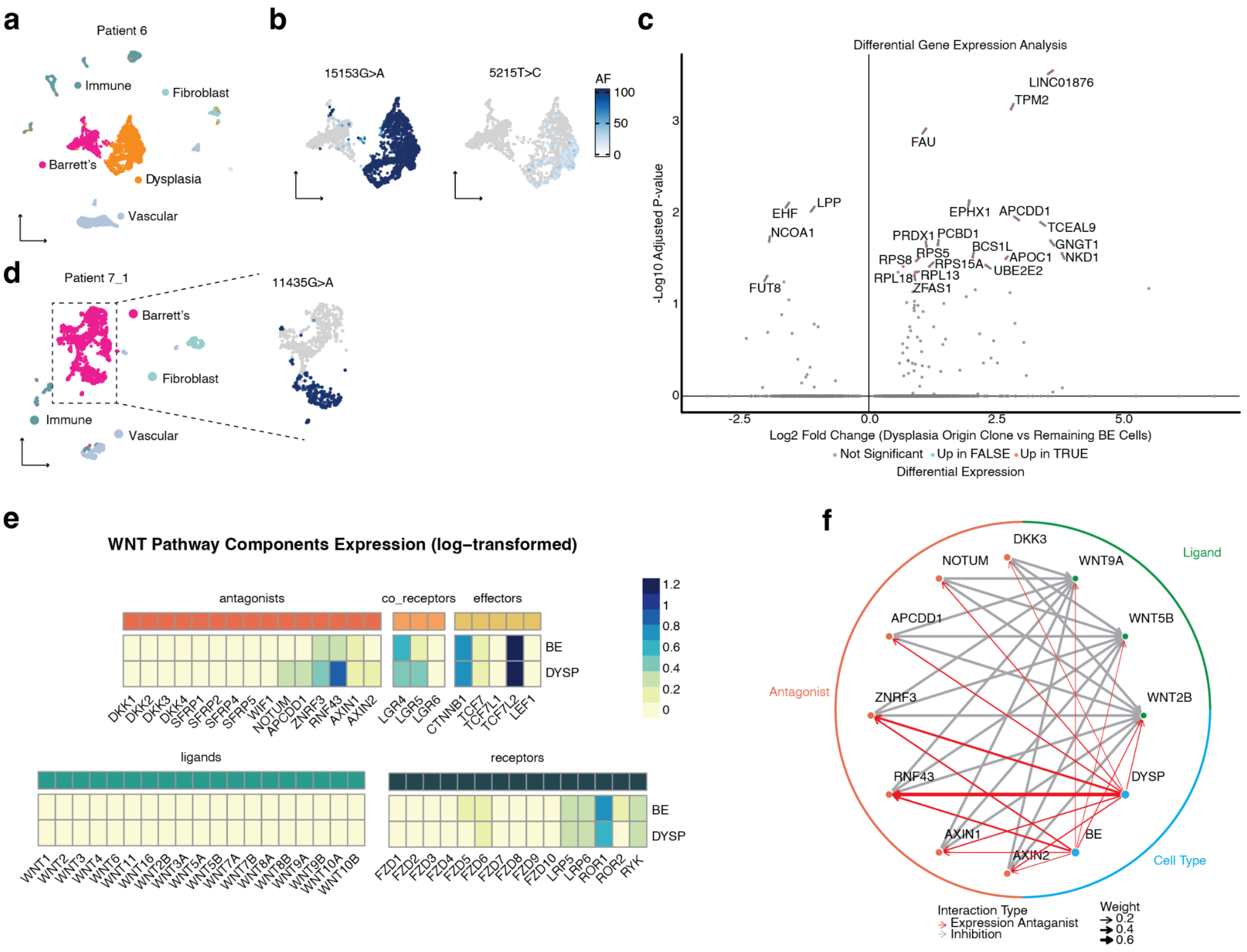

Fig. S18: Clonal and molecular characterization of dysplasia arising from Barrett's esophagus. (a) UMAP visualization of single-cell RNA-sequencing data from Patient 6 showing the different cell type clusters. **(b)** Allele frequency visualization of two key mitochondrial DNA mutations (15153G>A and 5215T>C) within the same cell populations, revealing clonal and subclonal relationships between dysplastic and non-dysplastic cells. **(c)** Volcano plot showing the differential gene expression analysis between dysplasia origin clone and remaining Barrett's esophagus cells. Key differentially expressed genes are labeled, with ribosomal proteins and transcriptional regulators prominently represented. **(d)** Analysis in Patient 7, showing a Barrett's esophagus biopsy with the 11435G>A mutation concentrated in a specific subpopulation of cells, suggesting early dysplastic transformation. **(e)** Heatmap showing expression of WNT pathway components across Barrett's esophagus and dysplastic cells, organized by functional categories: antagonists, co-receptors, effectors, ligands, and receptors. Dysplastic cells show upregulation of both WNT antagonists and selected effectors. **(f)** Gene-gene interaction network from CellChat showing relationships between WNT pathway components in dysplastic progression. Red nodes represent antagonists, green nodes represent ligands, and blue nodes represent cell types.

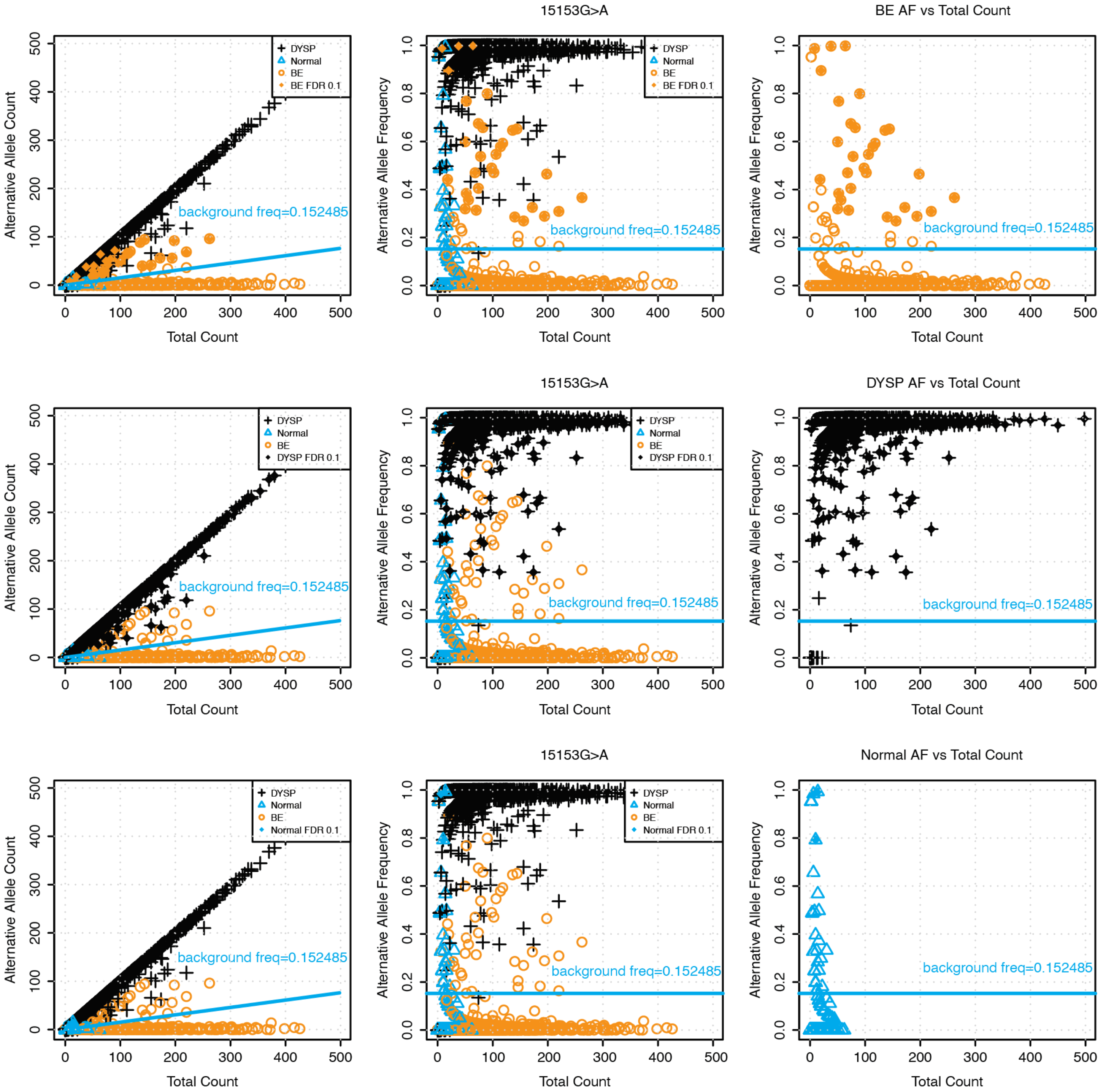

Fig. S19. ZIBB model applied to 15153G>A in Barrett's esophagus, dysplastic, and non-epithelial cell populations. Allele frequency profiles of mitochondrial variant 15153G>A in the cells from the Barrett's esophagus biopsy from Patient 6, plotted with the estimated background contamination rate in blue. The first column of plots shows the read counts for the alternative allele versus the total read counts at that position in all cells. The second column of plots shows the allele frequency versus the total read counts in all cells. The third column of plots is the same as the second column, only featuring a single cell type. Each row of plots highlights one of three cell types, Barrett's esophagus (BE) in the first, dysplasia (DYSP) in the second, and non-epithelial cells (Normal) in the third; highlighted cells were determined to be significantly different from background by the ZIBB model.

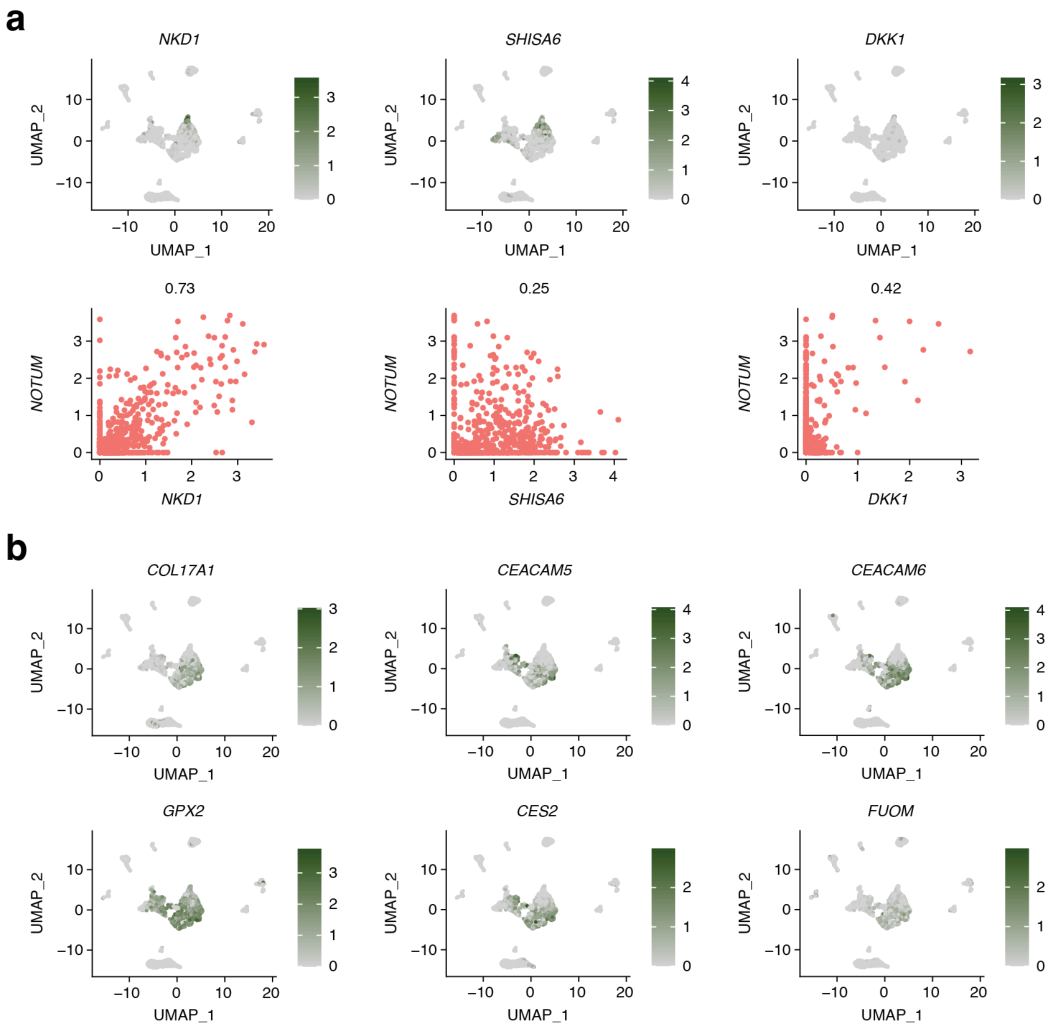

Fig. S20. Differential expression of WNT antagonists and potential fitness genes within dysplastic clones. (**a**) UMAPs of scRNA-seq of cells from a biopsy taken from a patient 6 with high-grade dysplasia examined in depth in Fig. 4 featuring differential expression of WNT antagonists within the dysplastic cells. Below the UMAPs are scatter plots of each gene with *NOTUM*, where the Pearson correlation coefficient is shown above. (**b**) UMAPs of the same sample as in (a) featuring the expression of genes differentially expressed in the dysplastic subclone marked by mtDNA mutation 5215T>C (see Fig. 4C).

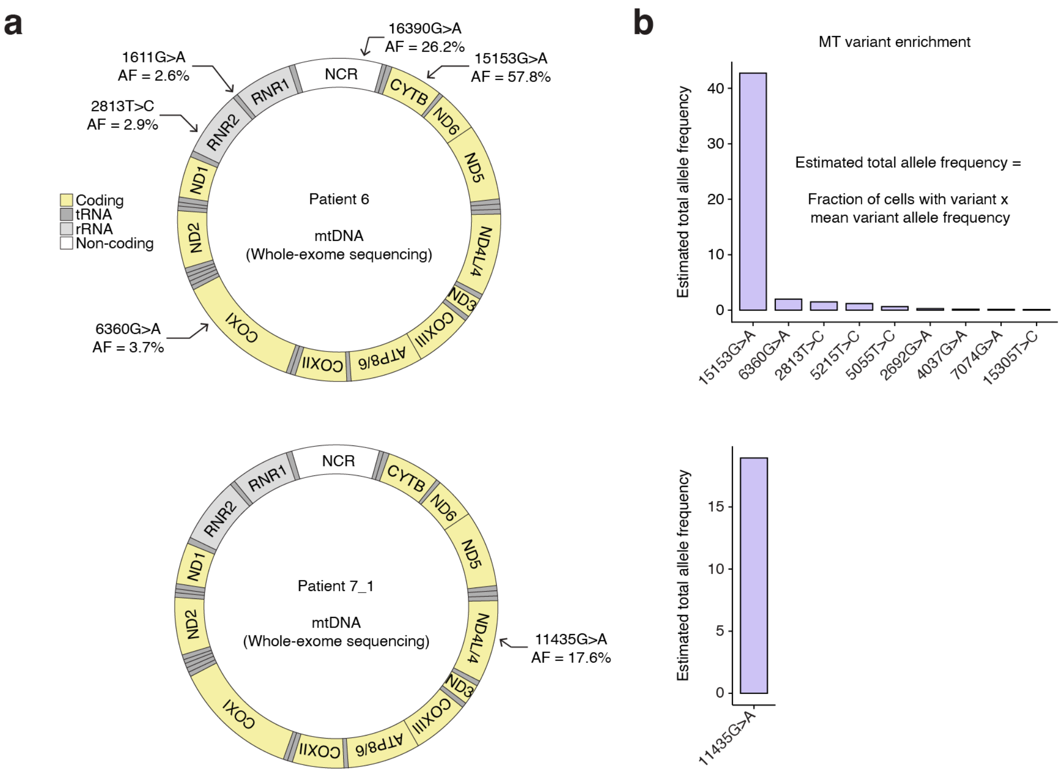

Fig. S21. DNA sequencing validation of mtDNA mutations detected with mitochondrial variant enrichment. (**a**) Schematic of the mitochondrial genome showing the mutations detected by whole-exome DNA sequencing in the two biopsies featured in Fig. 4; also displayed are their allele frequencies. (**b**) Barplot of the mitochondrial mutations detected in our single-cell analysis, with a "bulk" measurement of their allele frequencies within the entire sample.

patient no	patient ID	gender	diagnosis	BE lesion size	symptoms	PPI	treatment
1	A185	male	negative for dysplasia	C0M4	GERD	lansoprazole	N/A
2	A186	male	negative for dysplasia	C0M2	N/A	pantoprazole	N/A
3	A187	male	indefinite for dysplasia	C0M4	GERD	omeprazole	N/A
4	A188	male	negative for dysplasia	C0M1	N/A	omeprazole	N/A
5	A189	male	negative for dysplasia	C0M1	GERD	esomeprazole	N/A
6	A191	female	high-grade Barrett dysplasia	C4M4	GERD	N/A	underwent endoscopic submucosal resection at outside hospital
7	A192	male	low-grade dysplasia	C3M6	N/A	omeprazole	RFA
8	A193	male	high-grade Barrett dysplasia	C1M2	GERD	esomeprazole	EMR and RFA
9	A194	male	high-grade Barrett dysplasia	C0M1	GERD	pantoprazole	RFA
10	A195	male	negative for dysplasia	C1M2	N/A	prevacid	N/A
11	A196	male	negative for dysplasia	C0M2	GERD	omeprazole	N/A
12	A198	male	negative for dysplasia	C3M5	GERD	omeprazole	N/A
13	A199	male	indefinite for dysplasia	C1M3	GERD	esomeprazole	N/A
14	A190	female	negative for dysplasia	C2M5	N/A	pantoprazole	N/A
15	A197	male	negative	C0M2	N/A	omeprazol	N/A

			for dysplasia			e	
16	A200	male	negative for dysplasia	C1M2	GERD	esomeprazole	N/A

Supplementary Table 1. Patient demographics and clinical characteristics. Summary of the Barrett's esophagus patient cohort included in this study, including sex, endoscopic findings, histopathological diagnoses, and the number and types of biopsies collected for single-cell RNA sequencing and mitochondrial variant enrichment analysis.

Supplementary Table 2. Summary of mitochondrial variants detected across tissues. List of somatic mitochondrial DNA mutations identified through MAESTER and the ZIBB model, including variant position, nucleotide change, allele frequencies, number of cells containing each variant, and the tissue types in which variants were detected for each patient.

mtDNA mutation	patient no	BE	SQ	GC	TC	b/w biopsies	w/in biopsies
947T>C	3	X	X				X
2063G>A	3	X		X		X	
3047G>A	8	X	X			X	
9166T>C	9		X	X	X	X	X
11924T>C	9		X	X	X	X	
8119T>C	9		X	X			X
6258G>A	9		X	X			X
14484T>G	9		X	X	X		X
10363T>C	10	X		X			X
7236G>A	12	X		X		X	
2759T>C	12		X	X		X	
6150G>A	13		X	X	X	X	
