# Supplementary material for: Clonal cell states link gastroesophageal junction tissues with metaplasia and cancer
Source: Nat Commun. 2025 Dec 8;16:10952. doi: 10.1038/s41467-025-66302-w (PMC12686426; doi:10.1038/s41467-025-66302-w)
Supplement: Supplementary file 3 — Description of Additional Supplementary Files [file 41467_2025_66302_MOESM3_ESM.pdf]

<u>Description of Additional Supplementary Files</u>

Supplementary Data 1: Cell type annotations and marker genes. Complete list of cell types identified in Barrett's esophagus and gastroesophageal junction tissues with their defining marker genes, cell counts per patient sample, and corresponding cluster assignments from single-cell RNA sequencing analysis. The statistical test is a two-sided Wilcoxon Rank Sum test performed in Seurat.
