# Supplementary material for: Clonal cell states link gastroesophageal junction tissues with metaplasia and cancer
Source: Nat Commun. 2025 Dec 8;16:10952. doi: 10.1038/s41467-025-66302-w (PMC12686426; doi:10.1038/s41467-025-66302-w)
Supplement: Supplementary file 5 — Reporting Summary [file 41467_2025_66302_MOESM5_ESM.pdf]

nature portfolio

Corresponding author(s): Sydney M. Shaffer

Last updated by author(s): Sep 26, 2025

Reporting Summary

Nature Portfolio wishes to improve the reproducibility of the work that we publish. This form provides structure for consistency and transparency in reporting. For further information on Nature Portfolio policies, see our Editorial Policies and the Editorial Policy Checklist.

Statistics

For all statistical analyses, confirm that the following items are present in the figure legend, table legend, main text, or Methods section.

n/a	Confirmed	
☐	☒	The exact sample size (*n*) for each experimental group/condition, given as a discrete number and unit of measurement
☐	☒	A statement on whether measurements were taken from distinct samples or whether the same sample was measured repeatedly
☐	☒	The statistical test(s) used AND whether they are one- or two-sided<br>*Only common tests should be described solely by name; describe more complex techniques in the Methods section.*
☒	☐	A description of all covariates tested
☐	☒	A description of any assumptions or corrections, such as tests of normality and adjustment for multiple comparisons
☐	☒	A full description of the statistical parameters including central tendency (e.g. means) or other basic estimates (e.g. regression coefficient) AND variation (e.g. standard deviation) or associated estimates of uncertainty (e.g. confidence intervals)
☐	☒	For null hypothesis testing, the test statistic (e.g. *F*, *t*, *r*) with confidence intervals, effect sizes, degrees of freedom and *P* value noted<br>*Give P values as exact values whenever suitable.*
☒	☐	For Bayesian analysis, information on the choice of priors and Markov chain Monte Carlo settings
☒	☐	For hierarchical and complex designs, identification of the appropriate level for tests and full reporting of outcomes
☐	☒	Estimates of effect sizes (e.g. Cohen's *d*, Pearson's *r*), indicating how they were calculated

Our web collection on statistics for biologists contains articles on many of the points above.

Software and code

Policy information about availability of computer code

Data collection	10x GEM generation: 10x Chromium Controller. 10x and MAESTER library sequencing: Illumina NextSeq 550. WES library generation: Perkin Elmer Sciclone. WES library sequencing: Illumina NovaSeq 6000. Microscopy: Nikon Ti2-E.
Data analysis	10x demultiplexing and FASTQ generation: CellRanger mkfastq v5.0.0<br>10x alignment and count matrix generation: STARsolo v2.7.9a<br>10x doublet deconvolution: scDblFinder v1.8.0<br>10x dimensionality reduction, clustering and annotation: Seurat v4.0.2, Harmony v0.1.0<br>10x differentiation state analysis: CytoTRACE v0.3.3<br>MAESTER demultiplexing and conversion to FASTQ: bcl2fastq v2.20.0.422<br>Mitochondrial genome variant calling: maegatk v0.1.1<br>WES preprocessing and somatic variant calling: Picard v1.141, BWA MEM v0.7.10, GATK v4.2.5.0. IGV v2.12.3<br>WES copy number analysis: CNVkit v0.9.9<br>TCGA bulk transcriptome single-cell reference analysis: cellSignalAnalysis<br>Image analysis: NIS-Elements AR 5.20.02<br>Code for the ZIBB model is available on Zenodo under accession 17229078 [http://doi.org/10.5281/zenodo.17229078]. Diagnostic plots from ZIBB and FDR threshold analysis outputs are also available on the same accession. The code used for making figures has been deposited at Zenobo under accession 16997178 [https://zenodo.org/records/16997178].

For manuscripts utilizing custom algorithms or software that are central to the research but not yet described in published literature, software must be made available to editors and reviewers. We strongly encourage code deposition in a community repository (e.g. GitHub). See the Nature Portfolio guidelines for submitting code & software for further information.

Data

Policy information about availability of data

All manuscripts must include a data availability statement. This statement should provide the following information, where applicable:

- Accession codes, unique identifiers, or web links for publicly available datasets
- A description of any restrictions on data availability
- For clinical datasets or third party data, please ensure that the statement adheres to our policy

The single cell RNA raw sequencing data are deposited in the Gene Expression Omnibus (GEO) under accession number GSE291080 [https://www.ncbi.nlm.nih.gov/geo/query/acc.cgi?acc=GSE291080]. The spatial transcriptomics data are deposited in GEO under accession number GSE288235 [https://www.ncbi.nlm.nih.gov/geo/query/acc.cgi?acc=GSE288235]. The MAESTER data are deposited in GEO under accession number GSE287855 [https://www.ncbi.nlm.nih.gov/geo/query/acc.cgi?acc=GSE287855]. The previously published data compared in S14 from Nowicki et al., (2021) are available from the European Genome-phenome Archive (EGA) EGAD00001005438 [https://ega-archive.org/datasets/EGAD00001005438]. All raw microscope images are available on Zenodo under accession 17229323 [https://doi.org/10.5281/zenodo.17229323]. The whole exome sequencing data generated in this study have been deposited in the database of Genotypes and Phenotypes (dbGaP) under accession number phs003949. These data are available under restricted access due to patient privacy regulations and the presence of potentially identifying genetic information. To request access, researchers should submit a Data Access Request through the dbGaP Authorized Access system. Access requests must include a Data Use Certification signed by the investigator and institutional signing official, outlining the proposed research use and data security measures. The NIH Data Access Committee typically reviews requests within 2-4 weeks of submission. Once approved, access is granted for one year with the possibility of annual renewal, and data will remain available in dbGaP according to NIH data sharing policies.

Research involving human participants, their data, or biological material

Policy information about studies with human participants or human data. See also policy information about sex, gender (identity/presentation), and sexual orientation and race, ethnicity and racism.

Reporting on sex and gender	No sex- and gender-based analyses were performed. Barrett's esophagus is known to affect males disproportionately over females, but our study was not designed to investigate reasons for this disparity. Our study is also not sufficiently powered to differences in the disease states themselves between sexes, nor has this been commented on to our knowledge in the literature.
Reporting on race, ethnicity, or other socially relevant groupings	This information was not collected.
Population characteristics	Samples were collected from males (10) and females (2) with Barrett's esophagus ranging in age from 43-74.
Recruitment	Patients were recruited by G.W.F. and M.D. if they had a prior history of Barrett's esophagus but had not had surgery or ablation performed on the lesion. Patients consented to specimen collection.
Ethics oversight	Patient sample collection was approved by the University of Pennsylvania's Human Research Protection Program under IRB Protocol #813841.

Note that full information on the approval of the study protocol must also be provided in the manuscript.

Field-specific reporting

Please select the one below that is the best fit for your research. If you are not sure, read the appropriate sections before making your selection.

☒ Life sciences ☐ Behavioural & social sciences ☐ Ecological, evolutionary & environmental sciences

For a reference copy of the document with all sections, see nature.com/documents/nr-reporting-summary-flat.pdf

Life sciences study design

All studies must disclose on these points even when the disclosure is negative.

Sample size	Statistical analyses to determine an appropriate sample size were not performed. Single cells were dissociated from individual biopsies and processed according to established best practices in the field and budget constraints.
Data exclusions	Samples that consisted of cells pooled together from multiple biopsies were excluded from the mitochondrial variant enrichment analysis. No other samples were excluded. Doublets and low quality cells were filtered within each sample as outlined in the methods.
Replication	Barrett's esophagus cell types were observed in at least three of the four Barrett's esophagus patients in the 10x data. Furthermore, with the exception of ZMYND10, all in situ hybridization markers were observed in sections from at least two patients.
Randomization	Samples from patients were not randomized.

Blinding	Patients were de-identified by the clinical team and referred to by an identifier. Researchers only had access to basic demographic information and histological grading of different biopsies taken from the same lesion.

Reporting for specific materials, systems and methods

We require information from authors about some types of materials, experimental systems and methods used in many studies. Here, indicate whether each material, system or method listed is relevant to your study. If you are not sure if a list item applies to your research, read the appropriate section before selecting a response.

Materials & experimental systems

n/a	Involved in the study
☒	☐ Antibodies
☒	☐ Eukaryotic cell lines
☒	☐ Palaeontology and archaeology
☒	☐ Animals and other organisms
☒	☐ Clinical data
☒	☐ Dual use research of concern
☒	☐ Plants

Methods

n/a	Involved in the study
☒	☐ ChIP-seq
☒	☐ Flow cytometry
☒	☐ MRI-based neuroimaging

Plants

Seed stocks	N/A
Novel plant genotypes	N/A
Authentication	N/A
